# MAC-Bridging for Multi-PHYs Communication in BAN

**DOI:** 10.3390/s101109919

**Published:** 2010-11-05

**Authors:** Sana Ullah, Pervez Khan, Niamat Ullah, Kyung Sup Kwak

**Affiliations:** Graduate School of Telecommunication Engineering, Inha University, 253 Yonghyun-dong, Nam-gu, Incheon 402-751, South Korea; E-Mails: pervaizkanju@hotmail.com (P.K.); niamatnaz@gmail.com (N.U.); kskwak@inha.ac.kr (K.K.)

**Keywords:** WBAN, MAC, transparency, wireless, multiple, physical, frequency bands, channels, Body Area Network

## Abstract

Body Area Network (BAN) is a collection of low-power, miniaturised, and intelligent sensor nodes that are used for unobtrusive and ambulatory health monitoring of a patient without any additional constraints. These nodes operate on different frequency bands or Multiple Physical Layers (Multi-PHYs). Additionally, some BAN applications demand a logical connection between different nodes working on different Multi-PHYs. In this paper, the idea of controlling Multi-PHYs using one MAC protocol is introduced. Unlike existing procedures where different nodes working on different channels are connected at the link layer bridging/switching, the proposed procedure called bridging logically connects them at the MAC layer. In other words, the bridge is used to relay or filter packets between different PHYs in the same BAN. Numerical approximations are presented to analyze the stochastic behaviour of the bridges, all of them having Multi-PHYs interfaces. The MICS and the ISM bands are regarded as PHY1 and PHY2, respectively. The performance results are presented for PHY2 (given that data is already received from PHY1) in terms of probability of successful transmission, number of failed requests, power consumption, and delay. Simulations are conducted to validate the analytical results. It can be seen that the deployment of multiple bridges along with the corresponding nodes allows Multi-PHYs communication with high transmission probability, low power consumption, and tolerable delay.

## 1. Introduction

Body area Networks (BANs) consist of a number of smart and intelligent sensor nodes that are either implanted inside or connected around a human body to support a variety of medical and Consumer Electronics (CE) applications. Most of the applications include continuous health monitoring and extracting vital parameters of patients suffering from chronic diseases such as diabetes and cardiovascular diseases [1,2]. Like traditional sensor networks, nodes in BAN are extremely low-power and have limited communication range. But the nature of BAN is totally different from the traditional sensor network in terms of deployment, topology, network architecture, Quality of Service (QoS), scalability and interoperability. In BAN, there can be a number of nodes that work on different frequency bands and have correspondingly different Physical Layers/Channels (PHYs - Here, the whole frequency band is regarded as a single channel/PHY). Table 1 shows typical settings of BAN devices. The on-body nodes operate on Unlicensed Industrial, Scientific, and Medical (ISM) and Ultra wide (UWB) bands, while the in-body nodes operate on the licensed Medical Implant Communication Service (MICS) band (See [3]).

**Figure 1.**
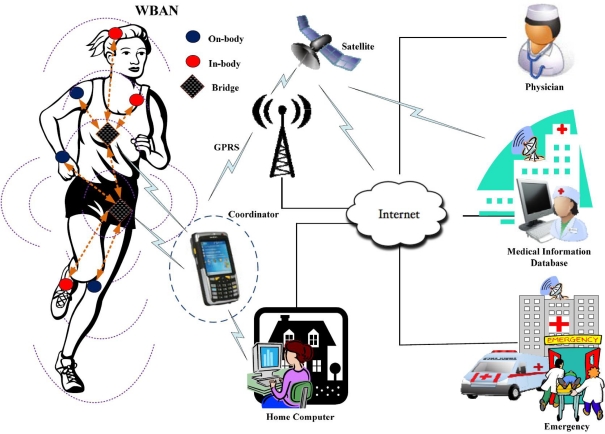
BAN architecture with multiple bridges.

One of the main networking challenges is how to connect different nodes working on Multiple PHYs (Multi-PHYs) in a single BAN. Since different nodes may adopt totally different access schemes, such synchronous or asynchronous access, centralized beaconing or distributed preamble transmission, their power consumption requirements differ from each other especially from the in-body nodes. Furthermore, data rates of these nodes vary from few Kbps to Mbps (on different PHYs) as given in Table 1. To accommodate all these demands at Multi-PHYs, a sophisticated mechanism should be integrated into the Medium Access Control (MAC) protocol for BAN. Traditional MAC protocols are designed for a single PHY only [4–6]. None of them consider the Multi-PHYs communication nor do they allow MAC layer connection between in-body and on-body nodes. This problem is identified as MAC transparency by the IEEE 802.15.6 [7], which is currently working on this issue. The early method of connecting nodes working on different PHYs includes link layer bridging/switching, provided that each PHY has a separate MAC protocol with independent MAC structure and control access methods [8]. However, the nature of BAN applications require one MAC (with the same MAC structure) to support Multi-PHYs simultaneously. In this paper, we introduce a new concept called bridging procedure that logically connects different PHYs at the MAC layer in a single BAN. The node implementing the bridging procedure, called the bridge, must have two or more PHY interfaces controlled by their corresponding MAC instances. Both on-body node(s) and the coordinator can be selected for the bridging due to their relatively larger capabilities and less stringent power requirements. The bridge relays or filters packets between different PHYs [9]. When many on-body nodes are selected for bridging, they need to contend for the resource allocation. Figure 1 shows a complete BAN architecture with multiple on-body bridges. The coordinator collects information from the nodes as well as the bridges and forwards it to a number of remote servers for diagnostic recommendations. We analyze the performance of multiple bridges with Multi-PHYs interfaces deployed in a single BAN as given in Figure 1. The MICS and the ISM bands are considered as PHY1 and PHY2, respectively. We are interested to see the probability of successful transmission and the average number of failed requests on PHY2. We further analyze the power consumption and delay of the whole BAN system integrated with multiple bridges.

The rest of the paper is categorized into four sections. Section 2 presents related works. Section 3 briefly describes the proposed procedure and presents useful analytical derivations. In Section 4, the performance of multiple bridges is analyzed. The final section concludes our work.

## 2. Related Works

Great research efforts are devoted to the design and development of a low-power MAC protocol for BAN. First, the focus remained on improving the IEEE 802.15.4 [10] and Bluetooth technology [11]. A significant improvement has been seen in the IEEE 802.15.4 in terms of superframe variation and contention access schemes [12–14]. The performance of beacon and non-beacon modes of IEEE 802.15.4 is investigated in [15,16]. The IEEE 802.15.4 has several drawbacks such as heavy collisions and retransmission. Moreover, it is not adaptable to traffic variation and is therefore not a suitable candidate for communication in BAN. The Bluetooth technology, on the other hand, has not received much attention because of its ineffectiveness and complexity. The contention-based protocols such as Carrier Sense Multiple Access/Collision Avoidance (CSMA/CA) is investigated in [17,18], where the authors concluded that CSMA/CA is not a reliable solution for BAN due heavy collisions and extra energy consumption. Most of the researchers considered Time Division Multiple Access (TDMA) architecture as a baseline for their protocols. In [19], the authors proposed a TDMA-based protocol, which exploits the static nature of BAN to implement effective TDMA strategies with little amount of overhead and no idle listening. A Reservation-based Dynamic TDMA (DTDMA) protocol is presented in [20], where the slots are allocated to the nodes which have buffered packets and are released to other nodes when the transmission is completed. Other protocols which are mainly based on TDMA architecture are presented in [21–23]. Zhou *et al.* proposed a BodyQoS protocol with an asymmetric architecture [24]. The BodyQoS has a unique property of adaptive resource scheduling, *i.e.*, in case the effective bandwidth of the channel degrades due to body fading effects, it adaptively allocates remaining bandwidth to the nodes. Another protocol called Distributed Queuing Body Area Network (DQBAN) is proposed for better QoS support in BAN [25]. Ghasemzadeh *et al.* proposed a power consumption technique that utilizes buffer information to reduce the number of transmissions among the nodes [26]. They further introduced compatibility graphs and described its importance in the power optimization [27]. The IEEE 802.15.6 is established for the standardization of low-power nodes in/around a human body to serve a variety of medical and non-medical applications. The standard is currently under development. Numerous MAC solutions are proposed to the standard committee in order to satisfy the requirements of BAN [28].

The above protocols are designed for a single PHY only. Furthermore, they consider a specific traffic model to address certain applications. None of them allow Multi-PHYs communication between nodes working on different PHYs in a single BAN. In the past we have connected different nodes working on different PHYs at the link layer since we might not need them to connect at the MAC layer. But in BAN, we need a MAC layer connection between nodes working on different PHYs and therefore we introduce the concept of bridging in this paper.

## 3. Bridging Description

The main concept behind the bridging function is to use a single MAC to support Multi-PHYs. Generally, nodes operating on MICS, ISM, and UWB bands have different MAC schemes for each band. The main problem is to connect these nodes under a single MAC. Here a single MAC means a common hybrid MAC framework which can be adopted in different PHYs. But the MAC parameters for each PHY may be different. This concept is similar to Class Definition and Objection Creation in the object oriented programming. The common MAC framework defines a MAC Class, and one MAC instance is created for each PHY according to its specific requirements. In other words, each PHY could have minor adjustments within the framework to meet its specific requirements. The common MAC framework uses a superframe based structure with hybrid multiple access mechanisms, *i.e.*, a combination of contention and scheduling access mechanisms.

**Figure 2.**
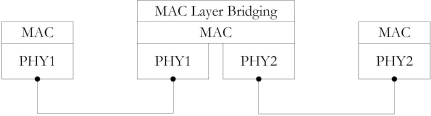
Bridging procedure: Protocol stack.

In the single MAC design, it is the bridging function that establishes logical connections between different nodes working on different PHYs. The node implementing the bridging function must have two or more different PHY interfaces. The necessary information from all the PHYs are recorded into a table called a bridging table. This table contains all the information regarding the BAN including PHY (MICS, ISM, and UWB) related information. The protocol stack of the bridging function is given in Figure 2. The bridge can collect or dissipate data from or to in-body/on-body nodes. As can be seen in the figure, the bridge has two PHY interfaces and can adapt the settings of both PHYs independently.

Both on-body nodes and the coordinator can be selected for the bridging purpose. In case the coordinator is selected, the resource allocation can be done via beaconing since coordinator controls the entire operation of the BAN. This is the most simple and ideal case of bridging. The coordinator (already controlling the operation on both PHYs) collects data from the source nodes and forwards it to the destination node. In case, the on-body nodes are selected for bridging, they need to contend for the channel in order to send/receive information to/from different PHYs. The following section presents a brief discussion on the superframe structure, resource allocation mechanism, and multiple bridges in a single BAN.

### 3.1. Superframe Interconnection

**Figure 3.**
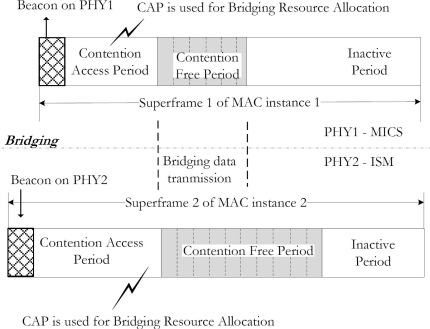
Bridging procedure: Two independent superframe structures.

The superframe structure of each MAC instance is the same but the size of active superframe including synchronization, clock frequency, and the beacon interval is independent. This is because the in-body nodes work with the low clock operation and they do not always require beacons to synchronize. As given in Figure 3, the superframe structures of both MICS and ISM bands are the same. They consist of a beacon, a Contention Access Period (CAP), a Contention Free Period (CFP), and an inactive period. But the protocol used in the CAP period may be different for each PHY. Furthermore, the active superframe period for in-body nodes is less than that of the on-body nodes since the in-body nodes have relatively longer sleep period. The bridge belongs to both PHYs at the same time and can accept their settings independently.

### 3.2. Resource Allocation

**Figure 4.**
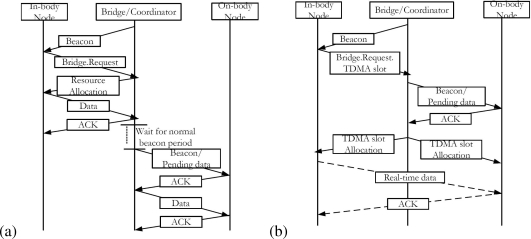
Coordinator as a bridge: **(a)** The bridge stores and forwards the packet, **(b)** The bridge establishes a real-time communication.

Initially the bridge records all necessary information including the network information, channel and node IDs, and the type of channel access mechanism into the bridging table. This table is used to relay or filter packets from the source node to the destination node. The in-body node, interested to send data to the on-body node, sends a bridging request to the bridge as given in Figure 4(a). The bridge allocates resources to the in-body node in order to receive the data (this is possible when the coordinator is used as a bridge. Otherwise, the bridge needs to contend for the resource allocation in the CAP period). Once the data is received, the bridge stores it and sends a beacon to the on-body node. The beacon informs the on-body node about the pending data. If the on-body node is ready to receive the pending data, it sends an Acknowledgement (ACK) packet and receives the data from the bridge. To support delay or jitter sensitive traffic, the bridge allocates the TDMA slots in the CFP period of both superframes on PHY1 and PHY2, respectively. Figure 4(b) shows the resource allocation process for a real-time traffic. The in-body node sends a request for the TDMA slot, the bridge first confirms if the on-body node is ready for a real-time transmission. Then the bridge allocates TDMA slots to both nodes. Once it is done, a real-time transmission can be started in the assigned time slots.

### 3.3. CAP Analysis for Multiple Bridges

Although the coordinator can be used as a bridge, some applications demand the use of multiple bridges in the same BAN. This can be done by allowing the bridges to contend for the resource allocation in the CAP part of the superframe. The contention protocol used in the CAP depends on the characteristics of each PHY. However, we consider the CSMA/CA protocol for both PHYs. Since the CAP is used by the corresponding nodes as well as by the bridges, the average delay of the bridged traffic (high priority traffic) is increased. One of the solutions is to use a small contention window for the bridges, which can reduce the average backoff time the bridge has to wait before transmission. Once the bridge receives data from PHY1, it contends for the resource allocation on PHY2 in order to send the data. The contention request can either be successful if the channel is idle or failed due to collision or successful transmission. Our interest is to analyze the probability of successful transmission and the average number of failed requests on PHY2, given that the data is already received from PHY1 (channel access mechanism on PHY1 is totally independent of PHY2, and thus traffic from PHY1 is regarded as arriving traffic to the bridge, which is forwarded on PHY2).

To derive analytical expressions, we define a stochastic process (*X*(*t*); *t* ≥ 0) which describes the stochastic behaviour of the bridge in the following way.

$$X(t)=\left\{\begin{array}{c} \mathrm{Idle}:\text{when}\;\text{the}\;\text{bridge}\;\text{is}\;\text{idle}\;\text{on}\;\text{both}\;(\text{all})\;\text{PHYs}\\ \mathrm{CCA}\;:\;\text{when}\;\text{the}\;\text{bridge}\;\text{is}\;\text{in}\;\text{CCA}\;\text{process}\\ \mathrm{BO}\;:\;\text{when}\;\text{the}\;\text{bridge}\;\text{is}\;\text{in}\;\text{backoff}\;\text{stage}\\ \mathrm{Tx}/\mathrm{Rx}\;:\;\text{when}\;\text{the}\;\text{bridge}\;\text{is}\;\text{transmitting}/\text{receiving} \end{array}\right\}$$

Now consider *n* number of bridges, all of them having Multi-PHY interfaces (both to PHY1 and PHY2), and they contend for the channel in the CAP period. The contention can be done either on one PHY or on both PHYs simultaneously. Let *r_n_* and *p_n_* be the transmission and conditional collision probability of any bridge in the network of *n* contending bridges, respectively. According to [29], *p_n_* can be derived as

(1) $$p_{n}=1-{\left(1-r_{n}\right)}^{n-1}$$

where *r_n_* can be derived from the average number of transmissions made by a bridge during the average backoff time to successfully transmit the data. The transmission probability *r_n_* is given by

(2) $$r_{n}=\frac{\bar{A_{n}}}{\bar{B_{n}}}$$

where $\bar{B_{n}}$ is the average backoff time for a bridge to successfully transmit the data when there are *n* contending bridges and $\bar{A_{n}}$ is the average number of transmission attempts made by a bridge during $\bar{B_{n}}$.

**Figure 5.**
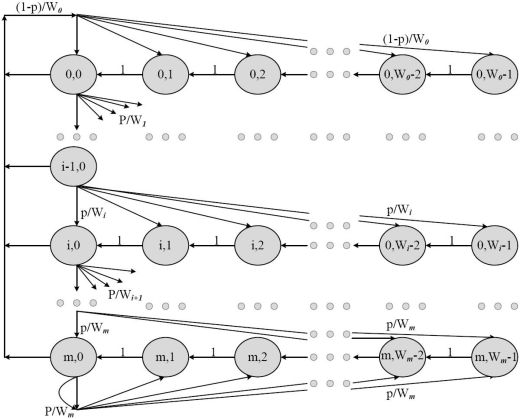
Markov chain model for the backoff window size.

For the expressions of $\bar{B_{n}}$ and $\bar{A_{n}}$, we first overview the exponential backoff procedure. The bridge intending to send a data frame waits for a period of time uniformly chosen from [0, *CW* −1] where *CW* is the contention window size. The backoff counter decreases by one for each idle time slot and stops in case of a busy channel. The bridge transmits the data frame whenever the backoff counter reaches zero. After a successful transmission, the value of *CW* is reset to minimum contention window size, *W = CW_min_*. If the transmitted data frame collides, it retransmits the data frame with a double backoff window size. The maximum backoff window size is *CW_max_* = 2*^m^W* where m is the maximum backoff stage. The state transition diagram of the CSMA/CA backoff procedure is given in Figure 5, where the notation (*i*, *W_i_*) shows the backoff window size of *W_i_* *=* 2*^i^W* at the *i*th backoff stage. Further details about the CSMA/CA backoff procedure including the transition probabilities are given in [29]. The average backoff time to successfully transmit the data is actually the average backoff time the bridge stays in each backoff stage as given in the figure. The final expressions for $\bar{B_{n}}$ is given by

(3) $$\begin{array}{l} \bar{B_{n}}=\sum_{i=0}^{k}p_{n}^{i}(1-p_{n})\left(\sum_{j=0}^{i}\frac{W-1}{2}.\sigma +(i+1).D_{\mathrm{CCA}}\right)\\ \;\;\;\;+p_{n}^{k+1}\left(\sum_{j=0}^{k}\frac{W\_1}{2}.\sigma +(k+1).D_{\mathrm{CCA}}\right) \end{array}$$

where *W* represents the contention window size, *k* represents the number of retries to transmit the data, σ is the length of the backoff slot, and *D_CCA_* is the time taken to perform CCA on the target channel. The size of contention window for the bridge should be less than that of the corresponding nodes. This approach reduces the average backoff time of the bridge before transmission. During the period of $\bar{B_{n}}$, a bridge makes $\bar{A_{n}}$ attempts to transmit a data frame, which can be modeled as a truncated geometric random variable according to [30] and is given by

(4) $$\bar{A_{n}}=(1-p_{n})+p_{n}(1-p_{n})2+\ldots \ldots \ldots .+p_{n}^{m}(k+1)=\frac{1-{\left(p_{n}\right)}^{k+1}}{1-p_{n}}$$

Now, in order to find the probability of successful transmission τ*_n_*, consider the probability that there is at least one transmission in a slot time, represented by γ*_n_*. The expressions for γ*_n_* and τ*_n_* are given as

(5) $$\gamma_{n}=1-{\left(1-r_{n}\right)}^{n}$$

(6) $$\tau_{n}=\frac{nr_{n}(1-p_{n})}{1-\gamma_{n}}$$

As mentioned earlier, we are interested to analyze the network performance by finding the probability of successful transmission (given by (5)) and the average number of failed requests on the targeting PHY. For *n* number of bridges, the successful transmission probability of a data packet after *k* retry limits is $1-(p_{n}^{k+1})$. Based on Equations (1) and (5), the conditional collision probability *p_n_* can be written as

(7) $$p_{n}=1-\frac{\tau_{n}(1-\gamma_{n})}{nr_{n}}$$

From the above expressions, the average number of failed requests *F_N_* in the CAP period is given as

(8) $$\begin{array}{l} F_{N}=N-\sum_{n=1}^{N}\left(1-p_{n}^{k+1}\right)\\ \;\;\;\;\;=N-\sum_{n=1}^{N}\left(1-{\left(1-\frac{\tau_{n}(1-\gamma_{n})}{nr_{n}}\right)}^{k+1}\right) \end{array}$$

where the second term of the equation represents the average number of successful requests, given that *N* requests are sent by the bridges.

## 4. Performance Evaluation

In this section, we present simulation setup and the results.

### 4.1. Simulation Setup

**Figure 6.**
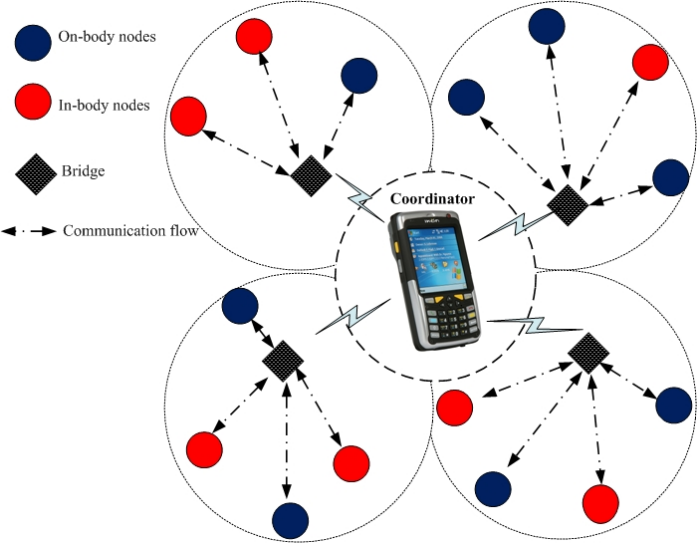
Simulation topology.

We investigate the performance of multiple bridges in terms of successful transmission probability, average number of failed requests, power consumption, and delay. Initially five bridges (including coordinator as a bridge) and 15 in-body/on-body nodes are considered in a single BAN. The simulation topology is given in Figure 6. The nodes use the nearest bridge to communicate with other nodes. Note that a direct communication between the nodes and the coordinator is always possible. The bridging facility is utilized whenever different nodes on different PHYs want to communicate. The in-body and on-body nodes operate on PHY1 and PHY2, respectively (the MICS and ISM bands are regarded as PHY1 and PHY2). All of the bridges have Multi-PHYs interfaces and are directly connected to the coordinator. Simulations are conducted using Monte Carlo method [31]. The bridge receives data packets from PHY1 and forwards them on PHY2. Poisson traffic is generated at the bridge (means that packets arrival at the bridge from PHY1 is a Poisson process).The data flow is considered from PHY1 to PHY2. Note that the following results are derived for PHY2, given that data is already received from PHY1. First, we are interested to analyze the probability of successful transmission and the average number of failed requests on PHY2. Then we analyze the average power consumption and delay of the bridges. During the CAP, *N* requests are sent by the bridges where $\sum_{n=1}^{N}\left(1-p_{n}^{k+1}\right)$ requests are successful and *F_N_* requests are unsuccesful. A request is dropped if the channel is busy, means that at least one bridge/node is transmitting. Note that the probabilities *r_n_* and *p_n_* are considered constant during each CAP, as done in [32]. The simulation parameters are given in Table 2, which are valid for PHY2 (2.4 GHz) only.

### 4.2. Simulation Results

**Figure 7.**
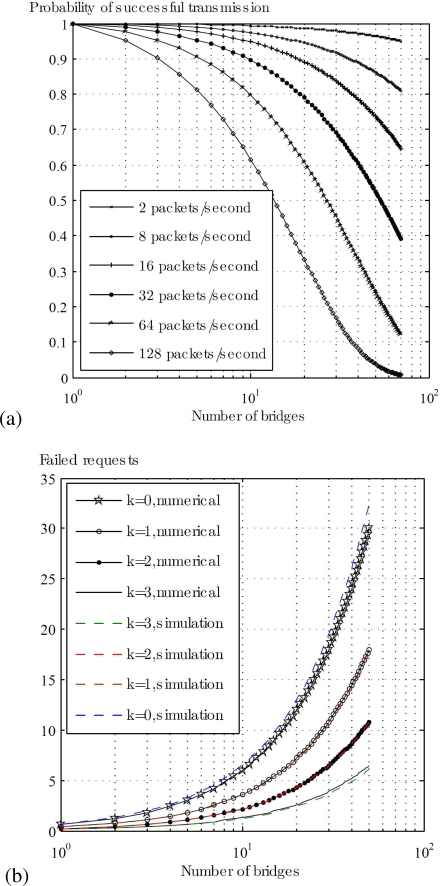
**(a)** Probability of successful transmission **(b)** Number of failed requests.

Figure 7(a) shows probability of successful transmission on PHY2 for different arrival rates from PHY1. For a low traffic load, the transmission is almost successful even if we deploy more than 50 bridges. But practically there can be at most 5 to 10 bridges in a single BAN, most of them having low traffic load (few applications require Multi-PHYs/real-time communication). In addition, if the traffic load increases up to 128 packets/second, the probability of successful transmission is approximately 0.9 for 5 bridges. Figure 7(b) shows the number of failed requests as a function of retry limits *k* and the number of bridges *n*. It can be seen that the number of failed requests increases with smaller *k* and higher *n*. For *k* = 0 and *n* = 50, the number of failed requests are almost 30, while for *k* = 3 and *n* = 50, the number of failed requests decreases to 7. As mentioned earlier, both the nodes and the bridges contend for the channel during the CAP, therefore the average number of failed requests increases due to collision and retransmission (if a request is dropped in the current CAP) in the CAP of the following superframe. Depending on the application, a separate and a dedicated slot(s) can be assigned to the bridges for contention. This will increase the successful number of requests since the nodes will not contend in the slot(s) dedicated to bridges.

**Figure 8.**
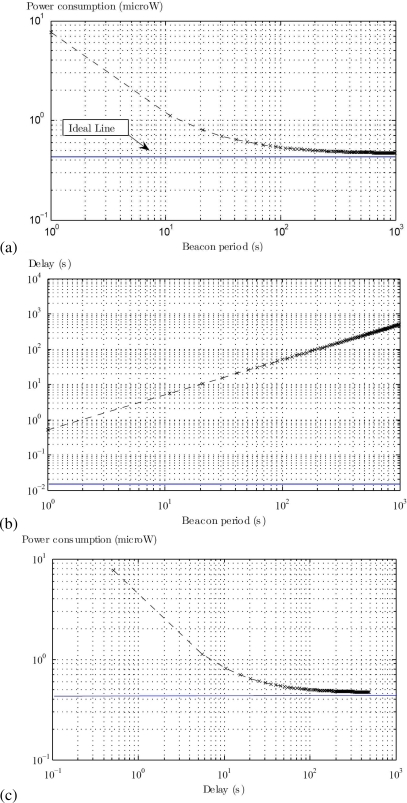
**(a)** Power consumption *vs.* Beacon period **(b)** Delay *vs.* Beacon period **(c)** Power consumption *vs.* Delay.

The average power consumption and delay of the bridges are given in Figure 8. Here, the idle state power is theoretically set to zero, as we intend to see the lower power limits of the bridges (indicated by ideal line). This can be used to optimize the power consumption parameters of the transceiver. Practically the idle state power must be considered to see the real performance. The packet arrival period is fixed to 1,000 seconds. The ideal line represents the ideal situation, where nodes grab beacons on the right time without any queuing delay. It can be seen in Figure 8(a) that the power consumption decreases as we increase the beacon period (interval between beacons). Moreover, for a long beacon period, packets have to wait longer before they are forwarded on PHY2. This increases the average delay of the system as given the Figure 8(b). By combing Figure 8(a,b), one can see a trade-off between the power consumption and the average delay for different beacon periods as shown in Figure 8(c). This can help the designer to optimize the value of the beacon period to achieve the desired results.

## 5. Conclusions

We presented the idea of a bridging procedure in order to connect Multi-PHYs in same BAN. Two PHYs, *i.e.*, the MICS and the ISM were considered and a logical connection between them were established. Monte Carlo simulations were conducted to analyze the performance of the bridges with Multi-PHYs interfaces in terms of successful transmission probability, number of failed request, power consumption, and delay. The proposed procedure supports MAC transparency by allowing multiple instances of the sameMAC protocol running on multiple processors for Multi-PHYs and their connection if required. Implementing the same MAC protocol on a single processor for Multi-PHYs encounters serious problems in terms of synchronization and clock operation, *i.e.*, to keep the same clock for all PHYs. However, the adaptation of the bridging procedure depends on the application requirements. It can be used if Multi-PHYs communication is required simultaneously.

## Figures and Tables

**Table 1.** In-body and on-body nodes requirements.

Device	Frequency Band	Data Rate	MAC	Power Supply	Application
On-Body (wearable) Device	ISM/UWB bands	up to 10 Mbps	CSMA, TDMA or a combination of both	High or Moderate Battery	Medical monitoring but could be used to connect different nodes
In-Body (implant) Device	MICS band	up to 500 Kbps	CSMA, Slotted ALOHA, TDMA or a combination of all	Limited Battery	Medical monitoring

**Table 2.** Simulation Parameters for PHY2 (2.4 GHz).

MAC Frame	100 bytes	Transmit Power	27*m*W
PHY Header	1 bytes	Receive Power	1.8*m*W
PHY Preamble	5 bytes	Idle state Power	5*μW*
Data Rate	250 Kbps	Setup Time	0.8*ms*
Beacon/ACK	10 bytes	Turn-around Time	0.4*ms*
*D_CCA_*	128 *μs*	σ	320*μs*
